# Emerging ideas and tools to study the emergent properties of the cortical neural circuits for voluntary motor control in non-human primates

**DOI:** 10.12688/f1000research.17161.1

**Published:** 2019-05-29

**Authors:** John F. Kalaska

**Affiliations:** 1 Groupe de recherche sur le système nerveux central (GRSNC), Département de Neurosciences, Faculté de Médecine, Université de Montréal, C.P. 6128, Succ. Centre-ville, Montréal (Québec), H3C 3J7, Canada

**Keywords:** cortical control of movement, representational models, dynamical neural networks, dimension reduction analysis, latent variables, neural manifolds, Ca++ reporter optical imaging

## Abstract

For years, neurophysiological studies of the cerebral cortical mechanisms of voluntary motor control were limited to single-electrode recordings of the activity of one or a few neurons at a time. This approach was supported by the widely accepted belief that single neurons were the fundamental computational units of the brain (the “neuron doctrine”). Experiments were guided by motor-control models that proposed that the motor system attempted to plan and control specific parameters of a desired action, such as the direction, speed or causal forces of a reaching movement in specific coordinate frameworks, and that assumed that the controlled parameters would be expressed in the task-related activity of single neurons. The advent of chronically implanted multi-electrode arrays about 20 years ago permitted the simultaneous recording of the activity of many neurons. This greatly enhanced the ability to study neural control mechanisms at the population level. It has also shifted the focus of the analysis of neural activity from quantifying single-neuron correlates with different movement parameters to probing the structure of multi-neuron activity patterns to identify the emergent computational properties of cortical neural circuits. In particular, recent advances in “dimension reduction” algorithms have attempted to identify specific covariance patterns in multi-neuron activity which are presumed to reflect the underlying computational processes by which neural circuits convert the intention to perform a particular movement into the required causal descending motor commands. These analyses have led to many new perspectives and insights on how cortical motor circuits covertly plan and prepare to initiate a movement without causing muscle contractions, transition from preparation to overt execution of the desired movement, generate muscle-centered motor output commands, and learn new motor skills. Progress is also being made to import optical-imaging and optogenetic toolboxes from rodents to non-human primates to overcome some technical limitations of multi-electrode recording technology.

## Introduction

For many years, neural recording studies of the cerebral cortical control of voluntary movements in awake, behaving animals were dominated by attempts to correlate the task-related activity of single neurons to the externally measurable properties of the executed movements. The development of simultaneous multi-neuron recording technologies and much more powerful computers over the past two decades has dramatically enhanced our ability to study cortical motor-control mechanisms. This has also led to translational applications such as brain–machine interfaces (BMIs) that allow non-human primates (NHPs) ^1– 6^ and paralyzed patients ^7– 11^ to impose real-time volitional control over computer cursors, robotic neuroprosthetic devices and even their own limb muscles ^12^ to perform various tasks. The focus of this review, however, is on how recent advances in quantitative tools to analyze population-level activity patterns are providing new insights into the cortical mechanisms of motor control and motor learning.

## Representational models of voluntary motor control

When single-electrode neurophysiological studies of cortical motor control began in the 1960s ^13, 14^, the field was dominated by “representational” models of brain function, which assumed that the activity of single neurons explicitly expressed specific kinds of information, such as particular properties of a sensory input or motor output. Behavioral and theoretical studies suggested that the conversion of an intention to move into muscle-centered motor commands could be described formally as a sequence of sensorimotor transformations between combinations of sensory and motor-related signals in definable coordinate frameworks, culminating in the generation of a descending motor command ^15– 21^. Computational models of voluntary motor control assumed that the motor system explicitly planned and controlled the specific features of reaching movements over which we appear able to impose volitional control, such as their direction, endpoint, spatial trajectory, velocity and output forces ^15– 34^. Representational models of brain function predicted that those controlled properties of movements would be explicitly encoded in the time-varying discharge patterns of single neurons generated while the motor system performed the neural equivalent of solving sets of equations that defined the inverse sensorimotor transformations between desired movement properties and causal muscle activity ^22– 34^. Neural correlates of the controlled parameters and coordinate transformations therefore would be directly observable in the task-related discharge of single neurons and could be identified by analyzing their activity in different motor tasks. Each neuron’s activity should show a consistent correlation to a particular parameter at all times before and during a movement, and the cortical control of movement could be understood by piecing together the contributions made by each neuron.

This conceptual foundation motivated many studies that used a wide variety of tasks to try to identify the motor output parameters and coordinate frameworks expressed by neurons in different cortical motor areas, including the primary motor cortex (M1), dorsal premotor cortex (PMd), ventral premotor cortex (PMv), supplemental motor area (SMA), parietal cortex area 5 (PA5) and adjacent medial intraparietal cortex (MIP). These studies revealed important differences in single-neuron response properties and in the strength and timing of correlations with different motor output parameters both within and across cortical areas that presumably reflected the different roles played by each neural population in motor control ^22– 34^.

These findings were consistent with the representational perspective on the cortical mechanisms of voluntary motor control. Ultimately, however, they have not provided a consensus as to the identity of the controlled parameter(s) or coordinate transformations that are encoded in any cortical motor area. Reasons for this failure include non-stationary correlations between single-neuron activity and motor output parameters at different times before and during movement, overlapping ranges of properties among neurons in different cortical areas, and partial correlations of single-neuron activity with multiple motor output parameters, in part because different movement parameters are coupled through the laws of motion and limb biomechanics ^34– 38^. When applied in their most simplistic literal sense—single neurons that unambiguously encode a specific controlled parameter in a specific definable coordinate framework—representational models do not account satisfactorily for the complexity of neural activity during the planning and execution of movements.

## Parsing the emergent properties of dynamical cortical motor circuits by dimensionality reduction

Multi-electrode recordings of the simultaneous activity of many neurons have provided critical neural data to test hypotheses that regard the cortical motor system as dynamical neural circuits whose emergent properties accomplish the computations underlying the planning and execution of voluntary movements ^34, 38– 42^. Here, the term “emergent properties” refers to the computational features of a neural circuit that arise from the interactions among the neurons within the circuit. The input–output transformations that generate a movement emerge as collective properties of the interactions among neurons within the circuits.

This perspective suggests that one cannot fully reveal how cortical circuits control movements by determining single-neuron correlates with different movement properties. Instead, one should analyze the activity of neural ensembles to try to parse out the internal computational structure by which the circuit contributes to movement control. One can envisage that the activity of *n* recorded neurons comprises an *n*-dimensional “state space” in which each neuron’s activity forms one axis (dimension) of that space. The instantaneous activity of the entire recorded population occupies a specific point in that state space at a given moment. The activity generating a given movement traces out a trajectory in that *n*-dimensional space as time progresses. Furthermore, the activity of overlapping subsets of neurons contributing to the unfolding neural trajectory is correlated in different ways via shared input signals and via the synaptic interactions among the neurons in the circuit. Trying to understand what the circuit is doing just by quantifying every neuron’s discharge rate at every moment in time is intractable. Instead, a more efficient approach that is now being used is “dimensionality reduction” (DR) ^38– 48^. DR seeks sets of time-varying patterns of response covariation (“latent variables”) which are shared by many neurons in the population as well as the weightings that determine how much each neuron’s activity contributes to each latent variable. This reduces the entire *n*-dimensional neural activity space into a much more compact and tractable low-dimensional space of latent variables that account for the majority of the total variance of the neural activity and that shape the trajectory of neural population activity through state space. The extracted latent variables capture patterns in the statistical covariance structure of the neural population activity which arise while the cortical neural circuits perform the computations required to generate movements. Rather than trying to identify whether a movement parameter is “encoded” by a single neuron, DR parses the statistical covariance structure of population activity patterns to identify multi-neuron correlates of different computational processes.

DR techniques are diverse and include principal component analysis (PCA), independent component analysis, factor analysis (FA), hidden Markov models, Gaussian process factor analysis, linear discriminant analysis and “demixed” PCA (dPCA) ^43– 48^, and other state-space ^49^ and factor ^46, 47^ models. New methods continue to be developed ^47, 50– 53^. All of these methods try to reduce a cost function associated with the covariance structure of the neural activity ^41, 44– 46^. Critically, however, different methods make different assumptions about the statistical structure of the neural data and seek specific features in that structure while ignoring features that might be better captured by other methods ^45, 46^. Thus, the choice of DR method can impact the interpretation and conclusions drawn from a neural data set. Furthermore, some DR methods, such as PCA, are performed on multi-trial-averaged activity and so can be used to analyze data collected during sequential recording sessions using conventional single electrodes, whereas others are performed on multi-neuron activity recorded during single trials to probe circuit function ^45– 53^. The key innovation of all DR methods is that they extract task-related patterns of multi-neuron co-modulation of activity—the latent variables—that are not observable when each neuron’s activity is processed separately.

DR analyses have yielded a number of novel perspectives on long-standing questions about the cortical control of reaching movements. For instance, classic models of the reaction-time process assume that the onset of a voluntary movement is preceded by essential neural events that prepare the motor system to generate a desired movement before it can emit the motor commands to execute it ^22– 33, 54– 58^. This preparatory activity has been extensively studied in instructed-delay tasks in which subjects are first given an instructional cue that provides information about the intended movement, such as the spatial location of a reach target, and later receive a “GO” signal to make the movement. Many neurons in PMd and M1 show changes in activity during the delay period which vary systematically with the information provided by the cue, such as broad directionally tuned activity as a function of the intended direction, amplitude and speed of the reaching movement ^22– 34, 54, 59– 62^. Representational models presume that those preparatory neural events implement the sensorimotor transformations that calculate the desired properties of the movement and that the observed single-neuron activity expresses the planned properties of the intended movement ^22– 34, 54– 62^.

Initial DR studies suggested a different way to view those neural responses ^38– 44^. They showed that the preparatory activity of the recorded population occupied a local region within the total possible neural-activity state space, dwelled within that local volume for the duration of the delay period, and then transitioned into movement-execution regions of state space after the GO signal appeared ^38– 41, 43– 45^. The instantaneous location of the population activity within the preparatory state-space volume when the GO signal appeared was significantly correlated with the reaction time in each trial ^63, 64^. The preparatory states prior to different reaching movements occupied different regions of state space and the subsequent movement execution-related activity for each movement followed a different trajectory through state space after exiting the preparatory state ^38– 41, 43– 45^.

Strikingly, a subset of the latent variables extracted from neural activity in the caudal part of PMd during movement execution exhibited strongly rotating trajectories through certain dimensions of state space whose amplitude and phase varied systematically as a function of the initial preparatory state and physical properties of each movement ^38, 40, 41, 65^. Those state-space rotations could be simulated by a simple linear dynamical model, suggesting that neural circuits in caudal PMd possessed dynamical properties during movement execution. This is consistent with theoretical models and behavioral evidence that the motor system displays computational properties of a dynamical system ^66– 70^. A critical feature of a dynamical system is that the change in the system’s state at any given moment is determined by its current instantaneous state. This led to the hypothesis that the cortical motor system generates a desired movement by first establishing the corresponding initial preparatory state. Once released from that preparatory state, circuits in caudal PMd contribute to the generation of the motor command by evolving along a neural trajectory in state space pre-determined by the initial preparatory state and driven by its own internal dynamics ^38– 41, 71^. This suggests a biologically plausible mechanism by which the motor cortical circuits can implement computations that accomplish the equivalent of a coordinate transformation between desired movement properties and causal muscle activity ^34– 38^.

These findings suggested that the widely documented single-neuron response correlates with different motor output parameters such as preparatory activity that predicts the direction, speed and length of an impending movement ^59– 62^, or that correlates with muscle activity and other evolving properties of the movement during execution ^34^ are the local expression of those state-space changes occurring at the population level within the neural circuit ^38– 41, 72^. More recent DR studies have yielded further novel perspectives on the role of preparatory activity and the transition from the preparatory to the movement-execution state.

Classic reaction-time models assume that the initial preparatory neural events are obligatory and must be expressed not only during the delay period of instructed-delay tasks but also early in the reaction-time period of non-delayed tasks ^54– 58^. A single-electrode study that compared PMd activity in reaction-time and instructed-delay tasks found evidence consistent with this prediction but could not distinguish distinct preparatory and movement-execution discharge components in the temporally compressed reaction-time activity ^54^. A recent study that addressed this issue with DR tools found evidence that provided stronger support for that prediction of the classic model ^73^. The investigators identified latent variables in the preparatory neural activity during an instructed-delay period which were maximally orthogonal to some latent variables extracted from execution-related activity recorded after the GO signal. They then showed that the neural activity recorded during the reaction-time period of two different non-delayed tasks always passed through an activation state resembling the preparatory state of the instructed-delay task before transitioning to the orthogonal movement-execution region of state space.

A long-standing question is why the preparatory activity in PMd during the instructed-delay period does not generate overt muscle contractions and movements ^62, 74, 75^. DR analyses provide one possible explanation ^75^. They revealed that the activity state space occupied during instructed-delay tasks could be divided into regions that can generate muscle activity (“output-potent”) and regions that cannot (“output-null”). Preparatory activity in PMd during the instructed-delay period is in the output-null region of state space (that is, a “prepare-but-withhold-movement” state). It then transitions into the output-potent region after the GO signal to generate muscle activity ^75^. This was recently extended by a study that found that the preparatory and execution-related regions of state space in PMd/M1 are almost completely non-overlapping and nearly maximally orthogonal ^76^. This occurred because the overall pattern of discharge correlations between all pairs of neurons in the population during the delay period was very different from that during movement execution, even though many neurons were active during both trial periods. This suggested that the dynamical computational structure and resulting emergent properties of the neural circuits change dramatically and rapidly during the transition from preparatory to movement-execution regions of state space ^76^. This could explain how the same neurons can discharge during both preparatory and execution phases of a trial and yet not produce muscle activity during preparation.

An obvious next question is how the dynamical computational structure of the neural circuits changes during the transition from a preparatory state to a movement-execution state. A study using a dPCA analysis of the post-GO activity in a delayed reaching task ^65^ extracted several “condition-variant” latent variables related to the different reaching movements and fixed “condition-invariant” latent variables that were associated with all movements independent of their details. The two sets of latent variables were orthogonal to each other in the dPCA state space. The condition-invariant latent variables explained much more of the total activity variance than the condition-variant latent variables. Importantly, the condition-invariant latent variables appeared to capture the process by which the population transitioned from a stable output-null preparatory region of state space to a dynamical output-potent region of state space that generated the time-varying motor output commands ^65, 71, 76^. This suggests that the condition-invariant activity reflected an internal computational process within the neural circuit that drove the network’s dynamics towards a state that can initiate movement without contributing to the planning or control of any feature of the ensuing movement. Similarly, a PCA-based DR analysis of M1 neural activity while monkeys reached to and grasped four different objects in one of eight different target locations (for technical reasons, only 24 of the 32 unique object/location combinations were used) ^77^ found that the largest component of task-related neural variance was condition-invariant. In contrast, condition-specific activity accounted for only about a third as much of the task-related neural variance. Finally, some components of M1 activity might serve primarily to maximize the separation of neural trajectories for different movements that otherwise might approach and become “tangled”, resulting in undesired motor outputs ^78^.

The novel insight provided by these DR analyses ^65, 71, 76– 78^ is that a major component of the task-related activity in M1 reflects aspects of the overall structure of the task such as transitions between stable postures and movement, and may have important functional roles in those processes without contributing directly to the specification of the properties of the motor output. This discharge component had been essentially ignored in prior single-neuron analyses. In contrast, the neural modulations that correlated with specific parameters of the task and that had preoccupied the field for many years comprised a significantly smaller proportion of total task-related neural variance.

## Future directions

The study of population-level activity using DR techniques continues to advance to provide intriguing new perspectives on the cortical mechanisms of voluntary motor control. They suggest that the emergent computational properties of dynamical neural circuits may provide a mechanism by which the cortical motor system can implement implicitly such algorithmic formalisms as sensorimotor coordinate transformations that describe how sensory and central signals may be converted into motor output commands. They indicate that the activity of each neuron is only a local window on the underlying low-dimensional computational processes by which entire neural circuits generate movements ^34– 41, 72^. This does not mean, however, that those single-neuron responses are uninterpretable epiphenomena. These studies confirm rather than refute the descriptions of single-neuron responses revealed in previous studies, while providing new ways of interpreting their nature, origin and role. Furthermore, the fundamental questions that motivated previous single-neuron studies are still valid and largely unanswered. How does the motor system transform diverse signals about the current state of the external world and the peripheral motor system and about the subject’s own internal physiological, motivational, and cognitive state into motor commands to generate the appropriate movement in the current context to fulfill a particular goal ^34– 38^? How do these processes allow for the volitional control of different properties of a movement in different contexts, such as speed versus accuracy, straight versus curved reach trajectories ^79^ or similar reach trajectories of the hand through space performed while holding the arm and hand in different postures ^80^? What roles do different neural populations and different cortical areas play in these processes and how can one account for the known differences in task-related activity in different cortical areas in a given motor task ^22– 34^? We need to understand what population-level computational processes within and across cortical regions could produce those widely documented single-neuron response correlates and what that reveals about how each population and cortical area contributes to voluntary motor control.

So far, however, most DR studies of cortical motor control have focussed on circuit dynamics in caudal PMd and M1 and how they might contribute to the generation of muscle activity ^38– 41, 63– 65, 71– 78^. Studies must expand into other cortical motor areas and to other behaviors such as grasping actions of the hand. A recent comparative study of sensory versus motor areas ^81^ has shown the utility of this approach.

For instance, the latent variables extracted from reach-related activity in SMA do not show rotational dynamics ^82^. This indicates that the evidence for rotational dynamics found in caudal PMd ^38– 41, 65^ is not a trivial or inevitable result of the task or the DR analyses. They also indicate that the two regions make different contributions to the control of reaching. A deeper analysis of the latent-variable structure of the activity in the two regions could help to clarify the nature of those differences. Similar approaches might provide a deeper understanding about the known differences in the directional tuning of M1, PMd and PA5/MIP activity in tasks with different degrees of dissociation of the direction of gaze versus reach ^29, 30^ and how neural correlates of causal forces are far more prominent in M1 than PA5/MIP during reaching movements with external loads or in isometric-force tasks ^34, 83– 86^.

Two studies used demixed DR to compare the activity of hand grasp-related neurons in PMv and anterior intraparietal cortex (AIP) ^87, 88^. Their findings showed several parallels with the reach studies in PMd/M1 ^38– 41, 62– 65^, suggesting some common features in the low-dimensional computational structure of the neural mechanisms underlying both behaviors. For instance, they identified distinct preparatory and execution-related regions of state space. Neural activity followed different trajectories through latent-variable space during both grasp preparation and execution, depending on whether the monkeys performed precision-pinch or power-grip actions, on the spatial orientation of the grasp object, and on whether they used the hand contralateral or ipsilateral to the neural recording site. The findings identified condition-variant and condition-invariant latent variables in the neural activity; the latter accounted for most of the total activity variance and were more prominent during movement execution than preparation. Importantly, they also found differences in the properties of the latent variables in the two areas. For instance, the neural activation state showed more prominent time-dependent changes during preparation in PMv than in AIP, suggesting that PMv is more implicated than AIP in preparation for the increasingly imminent initiation of movement as the delay period progressed. Neural trajectories in AIP were more closely coupled to the spatial orientation of the grasped object independent of the grasping hand but were more strongly coupled in PMv to the laterality of the hand used. These differences suggest different but overlapping roles for PMv and AIP in the reach-to-grasp task that, both interestingly and reassuringly, are consistent with earlier studies of single-neuron properties in the same two areas ^28, 33^. Similarly, activity in M1 during a reach-to-grasp task contained condition-variant latent variables associated with object locations and identities ^77^. The level of neural modulation in latent variables associated with object location versus identity shifted progressively in time, so that object location correlates were strongest near the onset of reach and object identity modulations were progressively stronger later in the trial as the hand approached the objects and adjusted its configuration to grasp them.

These various findings also indicate that it should be very informative to extend DR from separate analyses of activity in each cortical motor area to the pooled activity patterns recorded simultaneously in multiple areas of the same monkeys in the same task and using the same DR methods. This might provide unique new insights into how movement-related information is transformed across the distributed cortical motor system during the planning and execution of voluntary movements. DR should also be used to parse out how higher-order cognitive and decision-making processes interact with motor preparatory and execution circuits to select the appropriate action to perform in a given behavioral context ^31, 32, 61, 89– 95^. For instance, one study ^95^ documented how dorsolateral prefrontal neural populations can simultaneously express both the predominant color and direction of colored-dot random-motion stimuli in separate latent variables but selectively use only the color or the motion direction of the stimulus to choose the direction of a saccadic eye movement in a given trial while discounting the other stimulus property. Finally, to enhance the power of new experiments, we also need more robust hypothesis-validation tools to assess to what degree DR techniques reveal truly novel emergent features of neural circuit processing or simply reflect prior known properties of single-neuron responses ^72, 96^.

## Latent variables, neural manifolds and motor learning

DR reveals that the covariance patterns of multi-neuron activity during the performance of typical motor tasks such as reaching in 2D and 3D physical space occupy a limited region—a “neural manifold”—of the full theoretically possible *n*-dimensional neural state space ^97– 99^. This neural manifold contains the intrinsic statistical structure (the latent variables) resulting from all the combinations of multi-neuron activity co-modulation patterns within the network which are sufficient to control the movements used in a given task. Recent DR studies that leveraged BMI technology suggest that the neural manifold also determines which motor skills are easy to learn and which are difficult.

In typical BMI tasks, subjects control the movements of an effector such as a cursor on a monitor or a robotic arm by volitional modulation of neural activity recorded by multi-electrode arrays in cortical motor areas ^1– 11^. A “decoder” algorithm translates the recorded activity into control signals for the effector. The unique experimental advantage offered by the decoder is that the recorded neurons are the sole source of its input signals, and the mapping between their activity and effector motions is completely defined by the decoder algorithm. Studies have used BMI tasks to document how subjects learn to control an effector through the decoder and how they alter neural activity patterns as they try to adapt to experimental alterations of the decoder mapping between neural activity and effector motions ^6, 100– 104^.

One study ^97^ used a BMI paradigm to assess the contribution of the neural manifold to motor learning. At the start of each session, the investigators recorded neural activity in M1 while monkeys controlled cursor motions with a familiar (“intuitive”) decoder and used FA to identify the latent variables within the intrinsic neural manifold associated with the intuitive decoder. The investigators then altered the decoder mapping in very specific ways so that the required compensatory changes in recorded neural activity either remained within the intrinsic manifold or had to explore regions of state space outside of the manifold. Within-manifold re-mappings maintained the contributions of each neuron to the latent variables but altered the mapping between the latent variables and cursor motions. This allowed the monkeys to use the familiar covariance patterns of the intuitive manifold but they had to associate them with different movements. Outside-manifold re-mappings altered the way that single neurons contributed to the latent variables but preserved the mapping between each latent variable and cursor motions. This required the monkeys to learn new multi-neuronal activity covariance patterns for each movement.

The monkeys showed considerable adaptation to within-manifold re-mappings over a few hundred trials in a single recording session but very limited ability to adapt to outside-manifold re-mappings within the same time frame ^97^. These results suggested that the low-dimensional latent-variable structure within the intrinsic manifold imposes important constraints on motor learning. A subsequent study probed more deeply how the covariance structure of the intrinsic neural manifold associated with the intuitive decoder influenced adaptation to within-manifold perturbations ^105^. The optimal solution would be to create a new multi-neuron covariance pattern for each reach direction, essentially a new set of latent variables. Instead, the monkeys tended to retain the latent-variable structure of the intuitive manifold and learned how to reweight and reassign different intrinsic latent variables to new reach directions when the decoder mapping was changed. This also could not be explained by redundancy in muscle activity patterns ^106^. This provided further evidence that the circuit dynamics responsible for the multi-neuron co-modulation patterns in the intrinsic manifold, not the activity of single neurons, are the basic computational mechanism of motor control ^97, 105, 106^. This is consistent with other findings that monkeys initially attempt to adapt to decoder perturbations by searching through the neural activity patterns associated with their natural motor repertoire ^6, 107^.

These results showed that the monkeys could not acquire outside-manifold solutions during a single training session. However, monkeys can adapt to an arbitrary decoder re-mapping if allowed to practice for several training sessions ^101, 102^. Importantly, this longer-term learning involved changes in both the independent and coordinated variance across neurons ^108^. This provided further evidence that the latent-variable structure of the intrinsic manifold allows for rapid motor learning when that structure is preserved but that it can be altered over longer time frames.

## Optical imaging and optogenetics

Neural data collected with chronically implanted multi-electrode arrays have greatly enhanced our ability to study cortical function but this technology still has important technical limitations. Among them, they provide a very sparse sampling of a small fraction of all the neurons within the implanted cortical volume, the neurons are usually unidentified, and the number of isolatable neurons usually declines substantially over several months. Advances in electrode technology may resolve some of these limitations. For instance, newly developed Neuropixel probes carry about 1000 closely spaced recording surfaces on a shaft that is 1 cm long and 70 µm wide ^109, 110^. They can permit simultaneous observation of the activity of most or all of the hundreds or even thousands of recordable neurons along a long narrow cylindrical volume of neural tissue around the probe. The resulting 1– to 2–order of magnitude increase in the size of neural data sets will present new challenges and opportunities for data analyses ^47, 109, 110^.

However, a different potential solution to these limitations may be two-photon (2P) optical imaging of neural activity using fluorescent signals generated by Ca ^++^ reporter molecules expressed by neurons ^111– 113^ and optogenetic methods to modulate the activity of targeted neural populations ^114^. These techniques have developed rapidly in rodent and other small-animal models. 2P Ca ^++^ imaging allows the simultaneous observation of the spiking activity of most or all single neurons that express the Ca ^++^ reporter within a microscope’s field of view (FOV). One can reliably image the activity of the same identified neurons within the FOV for many weeks or months. One can locate each neuron within the 3D cortical volume and reconstruct the spatiotemporal pattern of activity within that cortical volume. Double-labelling of neurons with different markers can allow one to identify specific neural subpopulations within the FOV. One can examine cortical function from the macro level of hundreds to thousands of neurons to the micro level of single dendritic spines. Ultimately, one could link all of these observations about neural activity to computational models of cortical local-circuit function. These tools have been used successfully in rodent studies of motor control and motor learning, and DR has been used on those data to extract features of the computational structure of neural activity ^115– 126^.

The potential power of optical imaging and optogenetic tools has spurred interest in developing similar toolboxes for NHPs ^127– 131^. However, progress has been slow because of the lack of the many transgenic lines, promoters and other tools to manipulate gene expression in specific cell types that are available in rodent models. As a result, the field is still largely in the proof-of-concept stage. Nevertheless, successful imaging of fluorescent signals from populations of identified neurons for weeks and months has been demonstrated in several NHP species, including macaque monkeys ^128, 132– 139^, squirrel monkeys ^113^ and marmosets ^140– 143^.

Optical-imaging studies of primary visual cortex (V1) in NHPs have successfully reconstructed the functional organization within an FOV, including visual-stimulus orientation bands and other known features of V1 hypercolumn structure ^134, 135^. They have shown that single layer 2/3 V1 neurons are preferentially activated by relatively complex features of arbitrary visual stimuli such as curvature, junctions and corners ^136^ and that the representation of complex natural visual scenes is extremely sparse in layers 2/3 of V1 ^137^. Finally, the activity of 150 to 250 neurons has been imaged in M1 of marmosets while they performed reaching movements or attempted to adapt to external force fields during reaching ^143^. These initial results confirm the potential of 2P optical-imaging methods to study the activity of large populations of identified neurons within an FOV in the cerebral cortex of behaving NHPs over extended periods of time.

Nevertheless, current 2P optical-imaging methods have a number of important limitations. They provide signals about neural spiking activity but not local field potentials. Most Ca ^++^ imaging methods with the spatiotemporal resolution needed to observe the spiking activity of many single neurons can image to a depth of only about 1000 µm, so that all NHP imaging studies to date have been limited to cortical layers 2/3. Advances in Ca ^++^ reporter molecules and imaging optics might eventually extend the depth of imaging ^129, 131, 144– 146^, but the light scattering and absorption properties of the heavily myelinated cortical tissue of NHPs present a major technical challenge. Moreover, many important functional areas are buried deep in the sulci of NHPs with gyrencephalic brains, making them inaccessible to direct optical imaging from the cortical surface. Optically refractive GRIN (gradient-index) lenses or periscope-like mirror probes could be inserted into the sulcal folds, but they are relatively large (>1 mm), can cause tissue damage when inserted, and are too rigid to be appropriate for long-term chronic recordings ^147^. Very thin micro-endoscopes that can be inserted to any depth are under development ^147^.

Furthermore, the FOV of most optical-imaging studies of multi-neuron spiking activity is relatively small, typically ranging from 500 × 500 µm to 850 × 850 µm, and much less when imaging single dendrites or spines. An FOV smaller than 1000 × 1000 µm will image the activity in only a very small part of the entire M1 motor map for the arm or about one visual hypercolumn in V1 of a macaque monkey. This severely constrains the ability to study functional organization over large expanses of a given cortical region. New advances in microscope optics and scanner engines may substantially increase the FOV to dimensions more appropriate for brains of the size in NHPs ^148– 150^.

Finally, microelectrode recordings of well-isolated neurons provide streams of discrete action potentials that can be measured with millisecond precision. In contrast, the fluorescent optical signals are noisy and indirect signs of neural spiking activity, and measurement and estimation errors are introduced at every stage in the data acquisition and processing pipeline from signal generation to signal measurement and subsequent analysis. The optical-imaging system can observe only the fluorescent photons that happen to enter the optical aperture of the photo-multiplier sensor as it raster-scans the FOV. The time course of the fluorescent response to a single spike is slow and prolonged compared with the causal action potential. As a result, the fluorescent signals generated by each spike of a high-frequency multi-spike discharge burst with short inter-spike intervals will sum and deconvolution techniques to reconstruct the causal spike sequence can introduce estimation errors. Fluorescent signals from neurons whose somata overlap visually in the FOV further confound the spike deconvolution process. Motions of the brain during scanning cause shifts of the positions of imaged neurons within the FOV, and techniques to co-register sequentially scanned images can introduce errors in single-neuron alignment across images and in the measurement of each neuron’s fluorescent signals across time. These and other sources of measurement and estimation errors may introduce a significant level of uninformative and even potentially misleading noise in the reconstructed multi-neuron activity patterns ^151^. This may compromise the ability of methods like DR, that analyze the covariance structure of multi-neuron activity patterns, to parse out the internal computational dynamics of local neural circuits, especially at a temporal resolution approaching that afforded by microelectrode recordings of neural spike trains.
